# Use of Materials Based on Polymeric Silica as Bone-Targeted Drug Delivery Systems for Metronidazole

**DOI:** 10.3390/ijms20061311

**Published:** 2019-03-15

**Authors:** Katarzyna Czarnobaj, Magdalena Prokopowicz, Katarzyna Greber

**Affiliations:** Department of Physical Chemistry, Medical University of Gdańsk, al. gen. J. Hallera 107, 80-416 Gdańsk, Poland; kczar@gumed.edu.pl (K.C.); mprokop@gumed.edu.pl (M.P.)

**Keywords:** amorphous materials, ordered mesoporous silica, sol-gel preparation, drug carrier

## Abstract

Mesostructured ordered silica-based materials are the promising candidates for local drug delivery systems in bone disease due to their uniform pore size and distribution, and high surface area which affect their excellent adsorption properties, good biocompatibility and bioactivity, and versatile functionalization so that their properties can be controlled. Ordered mesoporous silica (MCM-41 type) was synthesized by a surfactant-assisted sol-gel process using tetraethoxysilane as a silica precursor and hexadecyltrimethylammonium bromide as the structure-directing agent. Functionalized silica materials containing various types of organic groups (3-aminopropyl, 3-mercaptopropyl, or 3-glycidyloxypropyl groups) were synthesized by the post-grafting method onto pre-made mesoporous silica. Comparative studies of their structural characteristics, the surface mineralization activity and release properties for the model drug Metronidazole (MT) were then conducted. It has been found that porosity parameters, mineralization activity and adsorption/release of metronidazole from mesoporous channels of silica can be regulated using functional groups which are chemically bounded with an outer silica surface. The preferential mineral nucleation was found on negatively charged surfaces—MCM-41, and mercaptopropyl and glycidyloxypropyl functionalized silica (MCM-SH and MCM-epoxy, respectively) in simulated body fluid (SBF solution), as well as a sustained release of MT. In contrast to them, aminopropyl-functionalized samples (MCM-NH_2_) achieved a high MT release rate. These results confirm the potential of silica-based materials for local therapeutic applications (as drug carriers and bone substitutes) in bone disease.

## 1. Introduction

Currently, the most common ways of delivering drugs to the body are oral and parenteral administration. However, these manners have lower efficiency for some therapies, e.g., treatment of bone diseases/infections.

Therefore, local delivery systems are more effective for such types of disease. The desired carrier in the local treatment of bones diseases should demonstrate the possibility to simultaneously introduce in situ drug release in combination with the filling and regeneration of the tissue defect, for example to stimulate the growth of the natural tissue helping in its replacement or complementation [1,2,3].

The ordered mesoporous silica-based materials are the leading candidates in tissue reconstruction technology. Their characteristic features include a low susceptibility to bacterial colonization, and absolute resistance to corrosion, unlike metallic implants. Upon implantation, silica materials exhibit bioactive properties—by hydroxyapatite formation on a surface they are able to bond with the living bone, which makes them biocompatible. In addition, because of their high surface area, controllable pore size, narrow pore size distribution, thermal and mechanical stability and easy surface functionalization, they may be used as carrier designated for the controlled release of therapeutic drugs [4,5,6,7,8,9,10].

MCM-41 silica (Mobil Composition of Matter No. 41) is a mesoporous material with a hierarchical structure that was first developed by researchers at Mobil Oil Corporation and that was initially introduced as adsorbent and catalyst [11]. After the first utilization of mesoporous silica in delivering drugs by Vallet-Regı’ and co-workers [12], this material has also acquired a lot of interest in pharmaceutical applications. Therapeutic formulations of various compounds like anti-inflammatory agents (i.e., ibuprofen, metronidazole), antibiotics (i.e., vancomycin, amoxicillin, gentamicin) and anticancer agents (i.e., doxorubicin, carboplatin) have been developed using MCM-41 formulations [13,14,15,16,17,18,19,20].

Generally, mesoporous silica-based materials are synthesized via a template-directed method in the presence of a surfactant, which is either neutral or charged, and that serves as a structure-directing agent for the in situ polymerization of orthosilicic acid [11].

In the case of MCM-41, silica has a hexagonal arrangement with a pore diameter of 2.5 nm to 6 nm wherein cationic surfactants, in particular hexadecyltrimethylammonium bromide—CTAB, are used as templates. The most common silica source for the synthesis of MCM-41 is tetraethyl orthosilicate (TEOS) using the sol-gel process (called inorganic polymerization). It involves the hydrolysis and condensation of the TEOS monomers into a colloidal solution (sol), which acts as a precursor to the formation of the ordered polysilicate (≡Si−O−Si≡) network around the surfactant micelles [12].

In recent years some researchers have demonstrated the usefulness of functionalized ordered mesoporous silica materials for the adsorption release of drugs such as famotidine, amoxicillin, itraconazole, methotrexate, and so on [21].

The many, possible surface modifications of silica materials allow the precise control of surface chemistry to modify drug loading and release characteristics.

Surface modification of silica is achieved by reaction with organoalkoxysilanes. Organoalkoxysilanes form Si–O–Si bonds to the surface in a condensation reaction with the residual silanol groups on the silica surface. Usually, silica is functionalized with alkyl-/aryl, 3-aminopropyl-, 3-mercaptopropyl-, and glycidyloxypropyl-silanes.

Usually, functionalization of mesoporous silica can be accomplished by co-condensation, and by post-synthetic grafting. Since the organosilanes are introduced during the one-step synthesis procedure (co-condensation), the organic groups are generally more homogeneously distributed in the framework compared to material external surface-functionalized by a grafting method. However, in the co-condensation synthesis, the structurally different precursors having different hydrolysis and condensation rates lead to a decreased degree of ordered mesoporous structures, and a reduction in the pore diameter, pore volume, and specific surface areas compared to that synthesized via the grafting approach [22,23].

Thus, the objective of this paper is to study the physicochemical properties and mineralization potential of ordered silica-based materials to assess the usefulness of these types of material in the construction of bioactive implants to reconstruct the bone structure. Next, the loading and release of Metronidazole (MT)—a nonsteroidal anti-inflammatory drug acting in the diseased bone area—from obtained carrier matrices based on functionalized mesoporous silica were compared.

Therefore, a cetyltrimethylammoniumbromide (CTAB)-templated, mesoporous silica MCM-41 synthesized under basic conditions was chosen as support. Functional groups including 3-aminopropyl, 3-mercaptopropyl, and glycidyloxypropyl groups were used to functionalize the mesoporous MCM-41 via grafting approach to maintain a mesoscopic order while metronidazole was used as model molecule to investigate the surface-drug interactions, and to explain an effect of the structural differences between the carriers on in vitro drug release.

The obtained carriers were subjected to pre-formulation studies necessary for a thorough investigation of silica materials structure (e.g., porosity and surface area measurements through the Brunauer, Elmer and Teller method (BET) using spectroscopic tools such as Fourier transform infrared (FTIR), powder X-ray diffraction (XRPD) and electron microscopy (TEM and SEM) analyzes). Drug release studies were performed using a USP Type IV, comprising a UV-VIS spectroscopy for monitoring drug concentrations in the acceptor fluid.

## 2. Results and Discussion

### 2.1. Characterization of Functionalized Mesoporous Materials

**Figure 1:**
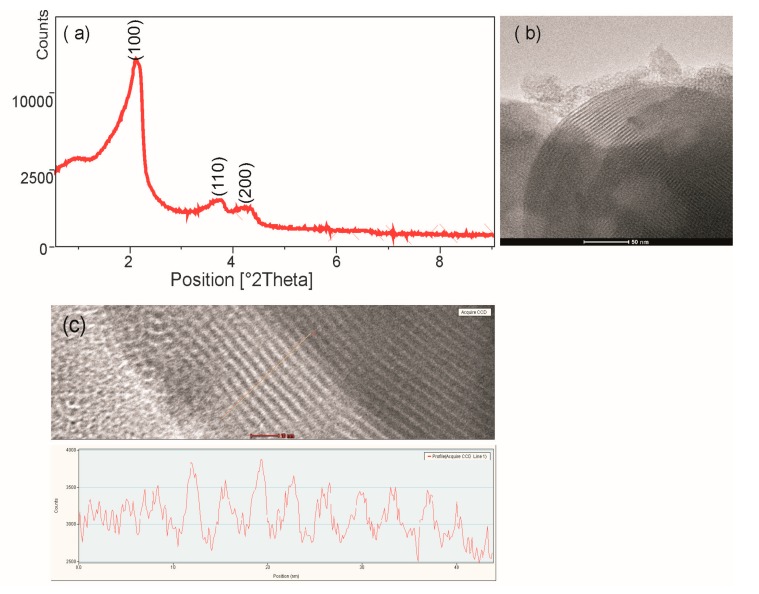
(**a**) X-ray diffraction pattern of MCM-41, (**b**) Transmission electron micrographs of MCM-41 in the (100) direction, (**c**) TEM measurement of the silica wall thickness and pore size.

Figure 1a shows the small-angle XRD pattern of MCM-41. The measurements of the silica sample revealed three diffraction peaks, which were indexed to the (100), (110) and (200) diffraction peaks confirming a highly ordered 2D-hexagonal mesoporous structure. Figure 1b shows the TEM image of the hexagonal arrangements of the porous system and the uniformity of the pores of silica. TEM measurement of the silica structure shows silica wall thickness ~5 nm, pore size ~3.3 nm (Figure 1c). The functionalized mesoporous materials also characterized by ordered mesostructures (not shown), they differ only slightly in pore size, which has been confirmed by BET studies (Table 1).

The mesoporous structures of MCM-41 and functionalized MCMs were investigated by N_2_ adsorption-desorption analyses. The specific surface area and pore sizes of the materials are reported in Table 1. All the samples showed high surface areas between 743–553 m2 g^−1^. The results reveal that these materials have average mesopore diameters between 2.93–3.51 nm and the pore volumes between 0.72–0.38 cm3 g^−1^. Among the samples tested, MCM-NH_2_ was characterized by smallest the specific surface areas, pore volumes and pore sizes.

**Figure 2:**
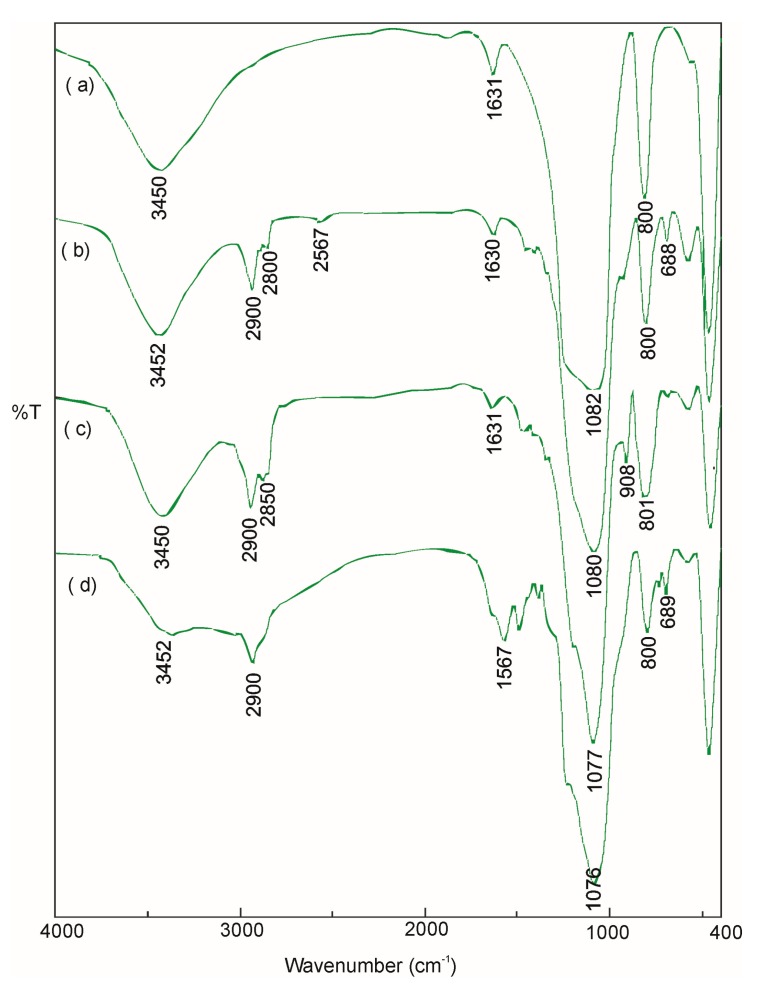
FTIR spectra of (**a**) MCM-41, (**b**) MCM-NH_2_, (**c**) MCM-SH, and (**d**) MCM-epoxy.

FTIR spectroscopy provided qualitative information about the chemical structure of the synthesized drug carriers. As shown in Figure 2 (spectrum a), no characteristic peaks of CTAB were detected, indicating the complete removal of surfactant from the pores of the synthesized MCM-41 material during the high-temperature calcination process. The resulting FTIR spectrum for the MCM-41 material showed bands derived only from bonds present in the silica. The strong bands were caused by the vibrations of Si–O–Si groups at 1076 and 800 cm^−1^, which is indicative of a high degree of polymerization. Two broad bands, one at 3450 cm^−1^ and the other at 1631 cm^−1^, were caused by O–H vibrations of Si–OH groups and the water molecule retained in the pores of the oxide network [24]. Figure 2 (spectra b, c, d) illustrate the FTIR spectra for functionalized MCM samples: MCM-NH2, MCM-SH and MCM-epoxy. Compared to the MCM-41, the all functionalized MCM samples exhibited additional absorption peaks of C–H bonds at 2900–2800 cm^−1^ region derived from alkyl chains of used organosiloxanes. For the MCM-NH2, additional absorption bands were observed at 1563 cm^−1^ and 689 cm^−1^, which were attributed to the vibration of N–H bonds. The MCM-SH exhibited characteristic absorption peaks of thiol group: S–H band at 2567 cm^−1^ and C-S band at 688 cm^−1^. For the MCM-epoxy, the peak at around 908 cm^−1^ was assigned to the vibration of C–O–C bonds. These observations demonstrated that organic groups were successfully grafted onto the MCM material [25,26].

### 2.2. Adsorption and Release of Metronidazole

The differences in surface area and pore size (Table 1) between the MCM-41 and three functionalized MCM samples cause the pure silica, and samples functionalized with mercaptopropyl and glycidyloxypropyl groups, to adsorb significantly more metronidazole than the samples functionalized with aminopropyl groups. This indicates that the porosity parameters (surface area, volume and pore size) are mainly responsible for differences in the adsorption capacity of materials.

The differences in adsorption properties of the samples to metronidazole could be partially attributed to the differences in the type of the functional groups and possible degree of interaction between the surface groups and MT molecules.

In the case of MCM-41, the surface of silica is negatively charged by the ionization of silanol groups in aqueous solution. The surface has an acidic character due to the possibility of proton displacement (-Si-O- + H+). Since metronidazole is a weak base that appears to have an affinity to the acidic surface of silica [27].

The silica surface modifications cause its hydrophobization but at the same time functionalization: -SH groups have the acidic character (stronger than -OH groups); -NH2 groups—basic character, the -epoxide groups easily open to the reactive diols, that are capable of complexing various compounds. The adsorption of MT appears to be more favorable on MCM-SH and MCM-epoxy carriers. The reduced adsorption of MT on MCM-NH_2_ carrier reflects the increase of repulsive forces between basic groups of surface and drug.

**Figure 3:**
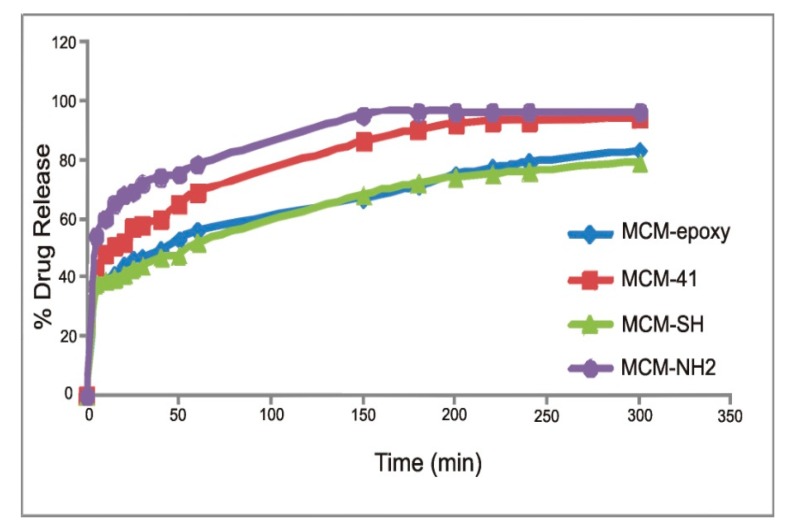
Mean cumulative release of MT (wt.%) from silica carriers during the 5 h of the experiment.

All obtained silica carriers with metronidazole have two-stage release profiles characterized by a faster initial release (0–10 min) followed by a constant slow-release. As shown in Figure 3, MT release from MCM-NH_2_ and MCM-41 was clearly higher compared to that from MCM-epoxy and MCM-SH. This study showed that 50% of the drug was released within the first 5 min from MCM-NH_2_, 15, 50 and 60 min from MCM-41, MCM-epoxy and MCM-SH, respectively. A complete release of the drug (95%) occurred within 2.5 hr from MCM-NH_2_, and 3.25 hr from MCM-41. In contrast, in the same time period only about 70% of MT from MCM-epoxy and MCM-SH was found to be released. A further release of MT from these carriers proceeded in a linear and slow manner. Complete amount of MT was released after 7 h and 7.2 h from MCM-SH and MCM-epoxy, respectively.

Based on the Korsmeyer-Peppas model [28], the release exponent, n, for the all drug-loaded carriers was between 0.40 and 0.53. These values are close to 0.5 and indicate a diffusion-controlled release.

This study shows that the type of organic modification of SiO_2_ determines the release rate of the drug. A much more rapid release was demonstrated in MT-MCM-NH_2_ system. The rapid release of the drug may be related to the smallest pore size and surface area of this material, therefore the lowest drug loading. MCM-NH_2_ samples holding low amounts of MT keep it on the outer surface of the matrix or close to the surface of the matrix and release it easily; however, release of the higher amount of MT from pure MCM-41 and MCM-SH and MCM-epoxy (having higher porosity parameters compared to MCM-NH_2_) takes longer and release does not occur easily. Another reason that may cause this phenomenon is the interactions between the drug substance and the matrix. The most important role in MT release is the hydrogen bonding between MT and the silica matrices. The MCM-41 has hydroxyl groups on the surface of the pores through which they can interact (through hydrogen bonds) with the drug and thus slow down its release. The addition of organic groups to the silica surface can cause changes in these interactions by: (i) increasing the hydrophobicity of the surface by the presence of alkyl groups; (ii) reducing the amount of surface hydroxyl groups as a result of the grafting reaction, and (iii) the surface functionalization through the presence of -NH2, -SH and epoxy-groups.

The addition of organosiloxanes hydrophobizes of surface and at the same time replaces the part of -OH groups with the alkyl groups which do not interact electrostatically with the drug; therefore, these matrices may have facilitated the release of the drug through its weaker interactions with drug. However, the hydrophobic carrier can hinder the penetration of the dissolution medium into the pores, which may slow drug release. Nevertheless, because this hydrophobization affects all carriers, it has no effect on the differences in drug release.

The functional groups, the most distant from the silica surface, most easily can interact with the drug molecule, causing its stronger binding in the pores (-SH and epoxy groups) or its repulsion (amine groups). The mentioned first can affect the slowdown of the release of the drug, in contrast to the amine groups, which may cause its easier release.

### 2.3. Evaluation of In Vitro Mineralization

The ceramic and glass oxide materials are considered bioactive if form a bone-like apatite layer on their surfaces after being implanted in bone. The ability of biomaterials to integrate with bone tissue can be evaluated using the simulated body fluid (SBF) test to study the in vitro formation of bone-like apatite at the surface of such materials when immersed in SBF [29]. SBF has almost equal compositions of inorganic ions to human blood plasma and does not contain any cells or proteins. In such an environment, bone-like apatite is created by the chemical reactions of the biomaterial components with the SBF ions. The mineralization activity of oxide materials decides both the chemical composition and the surface properties, especially the porosity (the presence of pores larger than 2 nm) and hydrophilicity of the surface [30]. These aspects suggest that ordered mesoporous silica materials, such as MCM-41 or functionalized MCM-41, thanks to high surface area, appropriate size of pores and the occurrence of reactive groups (-OH, -NH2, -SH, -epoxy), are the promising candidates as bioactive bone tissue substitutes.

To confirm the mineralization activity of the MCM-based materials, the samples of silica were soaked in SBF and then the formation of apatite phase was detected by FTIR, XRD, and SEM techniques.

**Figure 4:**
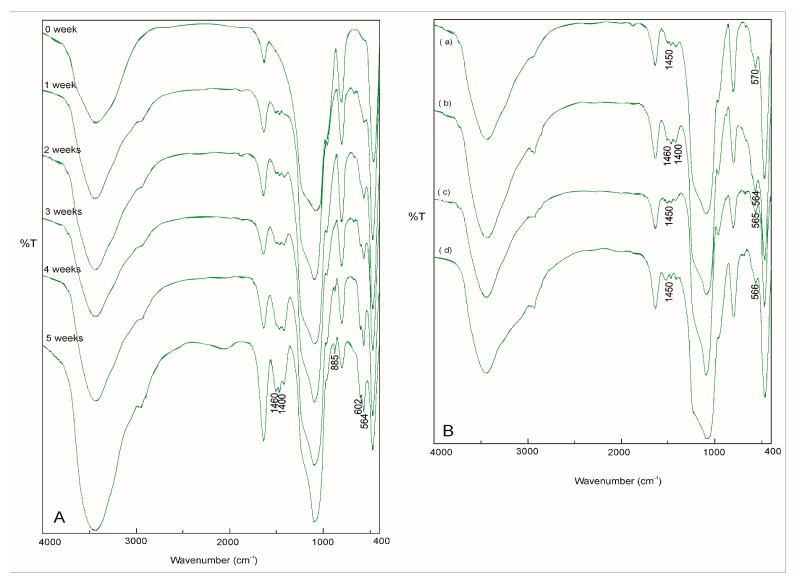
FTIR spectra of (**A**) MCM-41 during the 5 weeks of mineralization studies in simulated body fluid and (**B**) (a) MCM-41, (b) MCM-epoxy, (c) MCM-SH, and (d) MCM-NH_2_ after 2 weeks of mineralization studies in simulated body fluid.

The changes of FTIR spectra along with soaking time in SBF solution are illustrated in Figure 4B(a) taking MCM-41 as an example. After one week of experiment, the new absorption peaks appeared, and became stronger and stronger with longer soaking time. The existence of PO_4_^3−^ group was shown by the sharp doublet at about 564 cm^−1^ and 602 cm^−1^ wavenumbers (the major absorption mode of the phosphate groups—the O–P–O bending mode), and also 885 cm^−1^ (the P–OH stretching mode). In addition, carbonate groups CO_3_^2−^ can be detected in samples by the appearance of bands at 1400–1460 cm^−1^. Clearly, these peaks appearing in arrange of 500–1600 cm^−1^ indicates the formation of crystalline apatite with doping of some carbonate [31].

The induction period for the crystallinity of HA is different for various types of silicas. It was observed that the incubation of pure MCM-41 in SBF solution results in the fast growth of hydroxyapatite on its surface (1 week). In case of MCM-SH and MCM-epoxy, the appearance of HA on their surface was also after 1 week of the experiment, but the FTIR peaks were less intense. In case of MCM-NH2, the formation of HA on their surface was observed after two weeks of the experiment. In Figure 4B(b), the FT-IR spectra of all the four samples were put together and compared for apatite depositions after two weeks. The differences in the HA growth on the samples surface are related to its formation mechanism. Kokubo formulated a theory of the mechanism of in vitro apatite formation in bioactive materials (by immersion experiments in a simulated physiological solution—SBF) [32]. According to this, when silica-based material is soaked in SBF, a hydrated silica gel layer is formed. This layer, which is abundant in silanol (Si–OH) groups, provides favourable sites for the apatite nucleation, thanks to silanols complexation with Ca2+ ions. The absorbed Ca2+ ions may subsequently attract PO43- ions and form apatite layers on the surface of the material. This demonstrates that the surface functional groups, which are capable of binding Ca ions, may become sites for the surface nucleation. This applies especially to negatively charged groups (strong ion-ion interactions), followed by nonionic-polar (ion-polar interactions) and positively charged groups (repulsion of ions). The effective apatite formation ability observed for negatively charged group-bearing surfaces (with -OH, -SH and -epoxy groups) apparently proceeds by its strong ion-ion interactions with calcium ions and its subsequent phosphate ion adsorption.

Evaluation of the characteristic peaks of HA crystals by X-ray diffraction and the observation of surface morphology by SEM further confirmed the existence of HA.

**Figure 5:**
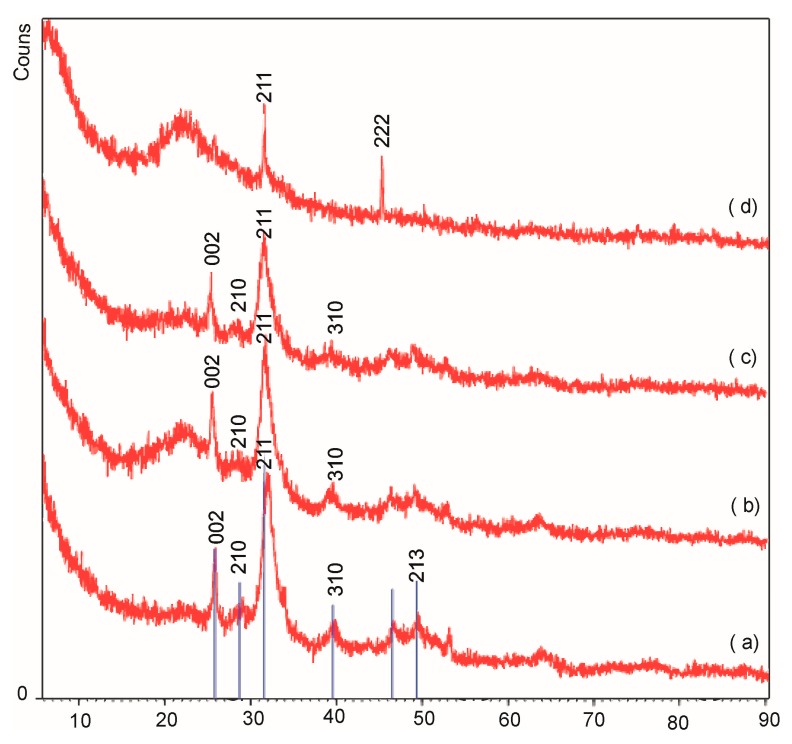
X-ray diffraction (XRD) patterns of (**a**) MCM-41 (**b**) MCM-epoxy, (**c**) MCM-SH, and (**d**) MCM-NH_2_ after 2 weeks of mineralization studies in simulated body fluid.

After in vitro mineralization experiment, the XRD analysis showed that all samples produced new peaks demonstrating the surface crystallization. The most intense peaks occur at 2θ 32º, 26º that are 2 1 1 and 0 0 2 diffractions respectively of the apatite (according to the standard JCPDS cards (09-0432)). As apparent from the diffraction pattern, the content of crystalline phase decreases for NH_2_-modified silica sample as shown by previous studies FTIR (Figure 5).

**Figure 6:**
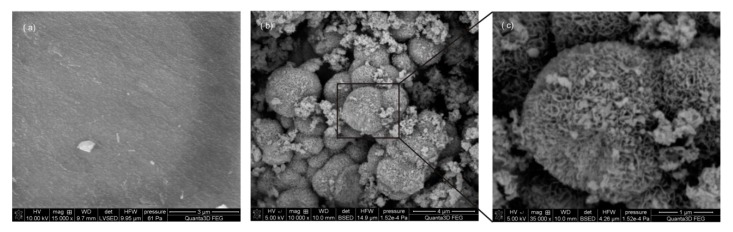
Scanning electron microscope (SEM) of (**a**) MCM-41 before mineralization studies, and (**b**,**c**) MCM-41 after 1 month of mineralization studies in simulated body fluid.

The formation of HA deposition was further proved by SEM analysis. Because of the similarity in SEM images, only images for MCM-41 sample was presented in Figure 6 to show silica surface before the experiment and then the deposited apatite on silica soaked in SBF for one month. Before the silica-based samples were soaked in SBF, all materials show smooth surfaces (Figure 6a for silica). After soaking for one month, the HA precipitates can be induced on the surfaces of all obtained types of silica. SEM images reveal that a large number of tiny plate-like crystals—characteristic for apatite—was deposited on the surface of these materials after mineralization experiment (Figure 6b,c).

## 3. Materials and Methods

### 3.1. Materials and Reagents

Tetraethoxysilane (TEOS), 3-aminopropyltriethoxysilane (APTS), 3-mercaptopropyltrimethoxysilane (MPTS), 3-glycidyloxypropyltrimethoxysilane (GPTMS), cetyltrimethyammoniumbromide (CTAB), tris(hydroxymethyl)aminomethane) (TRIZMA) were obtained from Sigma-Aldrich, Poznań, Poland. NaCl, NaHCO3, KCl, K2HPO4 3H2O, MgCl2 6H2O, CaCl2 2H2O, Na2SO4 hydrochloric acid (36.5%), ethanol (analytical grade purity), 25% aqueous ammonia and anhydrous toluene were purchased from POCh Co., Gliwice, Poland.

### 3.2. Synthesis of Functionalized Mesoporous Materials via Post-Grafting Method

First, MCM-41 silica was prepared and then functionalized with different organic groups.

MCM-41 was prepared according to the procedure reported by Grün et al. [33]. Typically, CTAB (2.39 g) was first dissolved in a polypropylene beaker containing a mixture of de-ionized water (125 g), 25% aqueous ammonia (9.18 g), and ethanol (12.5 g) for 1 h under stirring (200 rpm). Then, TEOS (10.03 g) was added dropwise with continuous stirring. After 2 h of stirring, the white precipitate was allowed to heat in an oven at 100 °C for 5 days. The obtained solid product was separated via filtration, washed with ethanol and water, and dried at 50 °C for 24 h. Synthesized material was calcined at 550 °C for 5 h (Muffle Furnace, M-525, USA, heating rate 1 K min^−1^) to remove the CTAB surfactant. Finally, silica polymer was obtained as a white powder.

The dry MCM-41 was grafted with 3-aminopropyl, 3-mercaptopropyl, and 3-glycidyloxypropyl groups by stirring 1.2 g MCM-41 with 4.4 mmol of APTS, MPTS, or GPTMS, respectively, in 200 mL toluene for 24 h. The selected MCM-41 to organosilane ratio is optimum for obtaining the maximum possible grafted groups in the materials, as reported by the scientific research [34]. The samples were then washed with a substantial amount of ethanol and dried at 100 °C.

### 3.3. Drug-Loading Efficiency

Metronidazole was loaded into MCM-41 by immersion with water solution of MT (the incipient wetness method) [35]. The drug loading experiment was performed by adding 50 mL of water solution of MT (1 mg mL^−1^) to a flask containing 1 g of MCM-41.

This mixture was shaken for 24 h at room temperature (25 ± 0.5 °C) under light-sealed conditions. Next, the supernatant was separated from the silica by centrifugation, and the residual MT content was determined using a UV–VIS spectrophotometer (Shimadzu UV-1800 spectrometer, Kyoto, Japan) by reading the absorbance at 320 nm. The percentage of drug adsorption versus initial concentration was plotted, allowing determination of the loading efficiency of the silica samples. The amount of absorbed MT in the sample was 18 mg MT g^−1^ MCM-41.

The loading of MT into organically modified silica samples was in a similar way as in the case of MCM-41. The loading efficiency obtained was 17, 20 and 11 mg MT g^−1^ sample, for MCM-epoxy, MCM-SH and MCM-NH_2_, respectively.

### 3.4. Metronidazole Release Experiments

Drug release studies were performed using the Flow-Through-Cell USP Apparatus 4 (closed loop) (Erweka, DZT-770, Heusenstamm, Germany). Typically, 100 mg of the carrier samples with drugs were placed on glass beads in a 22.6 mm flow-through cell. The SBF solution was used as an acceptor fluid. The experiment was carried out for 5 h in 100 mL of acceptor fluid with a flow rate of fluid of 4 mL min^−1^ at 37 °C.

At predetermined time intervals, 3 mL of release fluid was taken out for analysis of the release drug concentration with UV–VIS spectrophotometer, at λmax of 320 nm. Taken aliquot was replenished with fresh SBF (3 mL). All experiments were repeated in triplicate. Drug-elution data were plotted as the cumulative mass amount of drug released as a function of time.

The Korsmeyer-Peppas power law model was used as a mathematical description of drug release, expressed by the following Equation [28]

*M*_t_/*M*_∞_ = *kt*^n^ (1)

where *M*_t_/*M*_∞_ is a fraction of drug released at time *t*, *k* is the release rate constant, and *n* is the release exponent. The value of *n* characterizes the mechanism of drug release. The exponent *n* value close to 1 corresponds to zero-order release kinetics (the drug release rate is time-independent), *n ≤* 0.5 to a Fickian diffusion mechanism, and 0.5 < *n <* 1 to non-Fickian transport (the drug release is considered as anomalous). Generally, an early portion of a release profile (to 60%) is used in the Korsmeyer-Peppas model.

### 3.5. Characterization

The molecular structure of samples was determined using a Fourier transform infrared (FT/IR) spectrometer (410 Spectrometer, Jasco), using the potassium bromide (KBr) disk technique. The spectra were collected over a range of 4000–400 cm^−1^ (64 scans, resolution 4 cm^−1^).

The specific surface area and pore size distribution were determined by N2 adsorption using a Micromeritics ASAP 2405N instrument (Micromeritics, Norcross, GA, USA) and the Barret–Joyner–Halenda (BJH) methods, respectively.

The X-ray diffraction (XRD) spectra of samples were taken with an Empyrean/PANalytical XRPD diffractometer using CuKα1 radiation, operating at 20 kV and 40 mA.

The morphology of the silica was characterized using transmission electron microscope (TEM) (Tecnai G2 20X-TWIN, FEI Company, Hillsboro, OR, USA) and scanning electron microscope (SEM) (Quanta 3D FEG, FEI Company, Hillsboro, OR, USA).

### 3.6. Evaluation of In Vitro Mineralization

In vitro mineralization (ability to apatite formation on the silica surface) was performed as follows: 0.5 g of sample was incubated in 50 mL of SBF solution in the thermostated shaking water bath (Julabo, Seelbach, Germany, 50 rpm) at 37 °C (± 0.5 °C) for five weeks. The samples were tested daily using FTIR, XRD, and SEM techniques to detect apatite.

## 4. Conclusions

We studied the three important aspects of mesoporous silica implantable drug delivery systems: porosity, surface chemistry and ability for mineralization.

The results indicated that the functionalization of the silica surface significantly affects adsorption/release behaviour and mineralization activity, and also modifies the porosity parameters.

Among the tested carriers, pure silica and silica modified with MPTS and GPTMS have similar porosity parameters and the ability to mineralize the surface, in contrast to APTS modified silica, which exhibits smaller pores and a weaker ability for apatite nucleation. The modification of silica surface with SH and epoxy groups also influenced the slowdown of metronidazole release (compared to pure silica and especially silica modified with amine groups) due to the ability to create strong electrostatic interactions with the drug.

These results confirm the potential of silica-based materials for local therapeutic applications, as drug carriers and bone substitutes in bone disease.

## Figures and Tables

**Table 1:** Physical data of the silica-based materials.

Sample	Pore Diameter (nm)	BET Surface Area (m^2^ g^−1^)	Total Pore Volume (cm^3^ g^−1^)	MT Loading (mg MT g^−1^ Sample)
**MCM-41**	3.51	743	0.72	18
**MCM-epoxy**	3.44	659	0.57	17
**MCM-SH**	3.42	662	0.54	20
**MCM-NH_2_**	2.93	553	0.38	12
